# CAMSA: a tool for comparative analysis and merging of scaffold assemblies

**DOI:** 10.1186/s12859-017-1919-y

**Published:** 2017-12-06

**Authors:** Sergey S. Aganezov, Max A. Alekseyev

**Affiliations:** 1 Princeton University, 35 Olden St., Princeton, 08450 NJ USA; 2 ITMO University, 49 Kronverksky Pr., St. Petersburg, 197101 Russia; 3 The George Washington University, 45085 University Dr., Suite 305, Ashburn, 20147 VA USA

**Keywords:** Genome assembly, Assembly reconciliation, Scaffolding, Visualization, Breakpoint graph, Genome finishing

## Abstract

**Background:**

Despite the recent progress in genome sequencing and assembly, many of the currently available assembled genomes come in a draft form. Such draft genomes consist of a large number of genomic fragments (*scaffolds*), whose positions and orientations along the genome are unknown. While there exists a number of methods for reconstruction of the genome from its scaffolds, utilizing various computational and wet-lab techniques, they often can produce only partial error-prone scaffold assemblies. It therefore becomes important to compare and merge scaffold assemblies produced by different methods, thus combining their advantages and highlighting present conflicts for further investigation. These tasks may be labor intensive if performed manually.

**Results:**

We present CAMSA—a tool for comparative analysis and merging of two or more given scaffold assemblies. The tool (i) creates an extensive report with several comparative quality metrics; (ii) constructs the most confident merged scaffold assembly; and (iii) provides an interactive framework for a visual comparative analysis of the given assemblies. Among the CAMSA features, only scaffold merging can be evaluated in comparison to existing methods. Namely, it resembles the functionality of assembly reconciliation tools, although their primary targets are somewhat different. Our evaluations show that CAMSA produces merged assemblies of comparable or better quality than existing assembly reconciliation tools while being the fastest in terms of the total running time.

**Conclusions:**

CAMSA addresses the current deficiency of tools for automated comparison and analysis of multiple assemblies of the same set scaffolds. Since there exist numerous methods and techniques for scaffold assembly, identifying similarities and dissimilarities across assemblies produced by different methods is beneficial both for the developers of scaffold assembly algorithms and for the researchers focused on improving draft assemblies of specific organisms.

**Electronic supplementary material:**

The online version of this article (doi:10.1186/s12859-017-1919-y) contains supplementary material, which is available to authorized users.

## Background

While genome sequencing technologies are constantly evolving, researchers are still unable to read complete genomic sequences at once from organisms of interest. So, genome reading is usually done in multiple steps, which involve both in vitro and *in silico* methods. It starts with reading small genomic fragments, called *reads*, originating from unknown locations in the genome. Modern shotgun sequencing technologies can easily produce millions of reads. The problem then becomes to assemble them into the complete genome. Existing de novo genome assembly algorithms can usually assemble reads into longer genomic fragments, called *contigs*, that are typically interweaved in the genome with highly polymorphic and/or repetitive regions. The next step is to construct *scaffolds*, i.e., sequences of (oriented) contigs along the genome interspaced with gaps. The last but not least step is genome finishing that recovers genomic sequences inside the gaps within the scaffolds.

Unfortunately, the quality of scaffolds (e.g., exposing severe fragmentation) for many genomes makes the finishing step infeasible. As a result, the majority of currently available genomes come in a *draft* form represented by a large number of scaffolds rather than complete chromosomes [1]. This emphasizes the need for improving the assembly quality of genomes by constructing longer scaffolds from the given ones,^1^ which we refer to as the *scaffold assembly problem*. In other words, the scaffold assembly problem asks for reconstruction of the order of input scaffolds along the genome chromosomes.

A number of methods have been recently proposed to address the scaffold assembly problem by utilizing various types of additional information and/or in vitro experiments. These methods are based on jumping libraries [2–8], long error-prone reads (such as PacBio or MinION reads) [9–13], homology relationship between multiple genomes [14–16], wet-lab experiments such as the fluorescence *in situ* hybridization (FISH) [17, 18], genome maps [19–21], higher order chromatin interactions [22], and so on. Depending on the nature and accuracy of utilized information and techniques, assemblies produced by different methods may still be incomplete and contain errors, thus deviating from each other. Moreover, some scaffolding methods (e.g., based on FISH or HiC data) can produce assemblies, where the (strand-based) orientation of some assembled scaffolds is yet to be determined.

It therefore becomes crucial to determine what parts of different assemblies are consistent with and/or complement each other, and what parts are conflicting with other assemblies (or even within the same assembly). Furthermore, some scaffold assemblies may utilize only a fraction of the input scaffolds (e.g., homology-based assembly methods do not take into account unannotated scaffolds), thus posing a problem of analyzing and comparing assemblies of varying subsets of scaffolds. Comparative analysis of scaffold assemblies produced by different methods can help the researchers to combine their advantages, and highlight potential conflicts for further investigation. These tasks may be labor-intensive if performed manually.

While there exists a number of methods [23–29] for reconciling multiple assemblies of the same organism, they all are limited only to oriented scaffolds and thus are inapplicable to scaffold assemblies that include unoriented scaffolds. Furthermore, some of these methods require a reference genome sequence, which is often unavailable for non-model organisms. On the other hand, reconciliation methods that operate in de-novo fashion often process the input assemblies progressively, which makes such methods sensitive to the order of the input assemblies and affects the quality of the reconciled assembly.

We present CAMSA, a tool for comparative analysis and de-novo merging of scaffold assemblies. CAMSA takes as an input two or more assemblies of the same set of scaffolds and generates a comprehensive comparative report for them. Input assemblies can include both oriented and unoriented scaffolds, which enables CAMSA to process assemblies from the full range of scaffolding techniques (both *in silico* and in vitro). The generated comparative report not only contains multiple numerical characteristics for the input assemblies, but also provides an interactive framework, allowing one to visually analyze and compare the input scaffold assemblies at regions of interest. CAMSA also computes a *merged assembly*, combining the input assemblies into a more comprehensive one that resolves conflicts and determines orientation of unoriented scaffolds in the most confident way. The non-progressive nature of merging in CAMSA eliminates the dependency on the order of input scaffold assemblies. We remark that CAMSA can be utilized at different stages of the genome assembly process and be applied to assemblies of various genomic fragments, ranging from contigs to superscaffolds. In particular, CAMSA input can include results of other assembly reconciliation methods.

## Methods

### Assembly analysis and visualization

For the purpose of comparative analysis and visualization of the input scaffold assemblies, CAMSA utilizes the *breakpoint graphs*, the data structure traditionally used for analysis of gene orders across multiple species [30]. We will refer to the breakpoint graph constructed on a set of scaffold assemblies as the *scaffold assembly graph* (SAG).

**Fig. 1:**
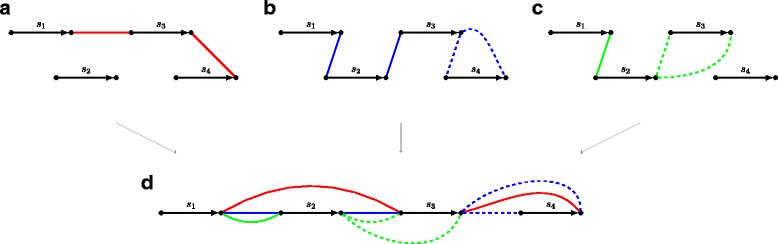
Individual scaffold assembly graphs for assemblies $A_{1} = \left \{\left (\protect \overrightarrow {s_{1}}, \protect \overrightarrow {s_{3}}, \protect \overleftarrow {s_{4}}\right), (s_{2})\right \}$ (red edges), $A_{2} = \left \{\left (\protect \overrightarrow {s_{1}}, \protect \overrightarrow {s_{2}}, \protect \overrightarrow {s_{3}}, s_{4}\right)\right \}$ (blue edges), and $A_{3} = \left \{\left (\protect \overrightarrow {s_{1}}, \protect \overrightarrow {s_{2}}, s_{3}\right), (s_{4})\right \}$ (green edges), and their scaffold assembly graph *SAG*(*A*_1_,*A*_2_,*A*_3_). Scaffold edges are colored black. Actual assembly edges are shown as solid, while candidate assembly edges are shown as dashed. **a** Individual scaffold assembly graph *SAG*(*A*_1_). **b** Individual scaffold assembly graph *SAG*(*A*_2_). **c** Individual scaffold assembly graph *SAG*(*A*_3_). **d** Scaffold assembly graph *SAG*(*A*_1_,*A*_2_,*A*_3_)

We start with the case of assemblies with no unoriented scaffolds. Then each assembly *A* can be viewed as a set of sequences of oriented scaffolds. We represent *A* as an individual *scaffold assembly graph* *SAG*(*A*) with two types of edges: directed edges (*scaffold edges*) encoding scaffolds in *A*, and undirected edges (*assembly edges*) representing scaffold adjacencies and connecting extremities (tails/heads) of the corresponding scaffold edges (Fig. 1 a, b, c).

We find it convenient to refer to each assembly edge as an *assembly point*. Equivalently, an assembly point in *A* can be represented by an ordered pair of oriented scaffolds. We specify the orientation of a scaffold *s*, either by a sign (+*s* or −*s*) or by an overhead arrow ($\protect \overrightarrow {s}$ or $\protect \overleftarrow {s}$). For example, $(\protect \overrightarrow {s_{1}}, \protect \overleftarrow {s_{2}})$ and $(\protect \overrightarrow {s_{2}}, \protect \overleftarrow {s_{1}})$ represent the same assembly point between scaffolds *s*_1_ and *s*_2_ following each other head-to-head. Clearly, any assembly is completely defined by the set of its assembly points.

To construct the scaffold assembly graph *SAG*(*A*_1_,…,*A*_*k*_) of input assemblies *A*_1_,…,*A*_*k*_, we represent them as individual graphs *SAG*(*A*_1_),…,SAG(*A*_*k*_), where the undirected edges in each *SAG*(*A*_*i*_) are colored into unique color. Then the graph *SAG*(*A*_1_,…,*A*_*k*_) can be viewed as the superposition of individual graphs *SAG*(*A*_1_),…,SAG(*A*_*k*_) and can be obtained by gluing the identically labeled directed edges. So the graph *SAG*(*A*_1_,…,*A*_*k*_) consists of (directed, labeled) scaffold edges encoding scaffolds and (undirected, unlabeled) assembly edges of *k* colors encoding assembly points in different input assemblies (Fig. 1 d). We will refer to edges of color *A*_*i*_ (i.e., coming from *SAG*(*A*_*i*_)) as *A*_*i*_-edges. The assembly edges connecting the same two vertices *x* and *y* form the *multiedge* {*x,y*} in *SAG*(*A*_1_,…,*A*_*k*_). The *multicolor* of {*x,y*} is defined as the union of the colors of individual edges connecting *x* and *y*.

We define the (ordinary) *degree* odeg(*x*) of a vertex *x* in *SAG*(*A*_1_,…,*A*_*k*_) as the number of assembly edges incident to *x*. We distinguish it from the *multidegree* mdeg(*x*) defined as the number of adjacent vertices that are connected to *x* with assembly edges.

When all assemblies *A*_1_,…,*A*_*k*_ agree on a particular assembly point {*x,y*}, the graph *SAG*(*A*_1_,…,*A*_*k*_) contains a multi-edge {*x,y*} composed of edges of all *k* different colors. In other words, both vertices *x* and *y* in this case have degree *k* and multidegree 1. For a vertex *z* in *SAG*(*A*_1_,…,*A*_*k*_), odeg(*z*)≠*k* or mdeg(*z*)≠1 indicate some type of inconsistency between the assemblies.

**Fig. 2:**
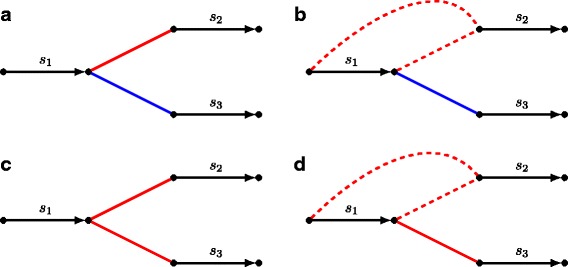
Illustration of various conflicts between assembly points of assemblies *A*_1_ (red edges) and *A*_2_ (blue edges). **a** Assembly points $\left (\protect \overrightarrow {s_{1}}, \protect \overrightarrow {s_{2}}\right)$ from assembly *A*_1_ and $\left (\protect \overrightarrow {s_{1}}, \protect \overrightarrow {s_{3}}\right)$ from assembly *A*_2_ are *out-conflicting*. **b** Assembly points $\left (s_{1}, \protect \overrightarrow {s_{2}}\right)$ from *A*_1_ and $\left (\protect \overrightarrow {s_{1}}, \protect \overrightarrow {s_{2}}\right)$ from *A*_2_ are *out-semiconflicting*. **c** Assembly points $\left (\protect \overrightarrow {s_{1}}, \protect \overrightarrow {s_{2}}\right)$ and $\left (\protect \overrightarrow {s_{2}}, \protect \overrightarrow {s_{3}}\right)$ both from *A*_1_ are *in-conflicting*. **d** assembly points $\left (s_{1}, \protect \overrightarrow {s_{2}}\right)$ and $\left (\protect \overrightarrow {s_{1}}, \protect \overrightarrow {s_{2}}\right)$ both from *A*_1_ are *in-semiconflicting*

For given scaffold assemblies *A*_1_,…,*A*_*n*_, we classify an individual assembly point *p*∈*A*_*i*_ representing an *A*_*i*_-edge {*x,y*} in *SAG*(*A*_1_,…,*A*_*k*_) as follows:

- **unique** if the multicolor of {*x,y*} is {*A*_*i*_}, i.e., the assembly point *p* is present only in a single assembly;
- **conflicting** with an assembly point *c*∈*A*_*j*_ if *c* represents an assembly edge {*x,z*} with *z*≠*y* (i.e., mdeg(*x*)>1), or an assembly edge {*y,z*} with *z*≠*x* (i.e., mdeg(*y*)>1). In particular, *p* is
  - **in-conflicting** with *c* if *i*=*j* (Fig. 2 c);
  - **out-conflicting** with *c* if *i*≠*j* (Fig. 2 a).^2^
- **non-conflicting** if *p* is not conflicting with any other assembly points.

We say that an assembly point is in-/out- conflicting if it is in-/out- conflicting with at least one other assembly point.

### Dealing with Unoriented scaffolds

While conventional multiple breakpoint graphs are constructed for sequences of oriented genes, in CAMSA we extend scaffold assembly graphs to support assemblies that may include oriented as well as unoriented scaffolds.

In addition to (oriented) assembly points formed by pairs of oriented scaffolds, we now consider semi-oriented and unoriented assembly points.

A *semi-oriented* assembly point represents an adjacency between an oriented scaffold and an unoriented one. For example, $(\protect \overleftarrow {s_{1}},s_{2})$ and $(s_{2},\protect \overrightarrow {s_{1}})$ denote the same semi-oriented assembly point, where scaffold *s*_1_ is oriented and *s*_2_ is not (as emphasized by a missing overhead arrow). Similarly, an *unoriented* assembly point represents an adjacency between two unoriented scaffolds. For example, (*s*_1_,*s*_2_) and (*s*_2_,*s*_1_) denote the same unoriented assembly point between unoriented scaffolds *s*_1_ and *s*_2_.

We define a *realization* of an assembly point *p* as any oriented assembly point that can be obtained from *p* by orienting unoriented scaffolds. We denote the set of realizations of *p* as *R*(*p*). If *p* is oriented, then it has a single realization equal to *p* itself (i.e., *R*(*p*)={*p*}); if *p* is semi-oriented, then it has two realizations (i.e., |*R*(*p*)|=2); and if *p* is unoriented, then it has four realizations (i.e., |*R*(*p*)|=4). For example,

$$R\left((s_{1},s_{2})\right) = \left\{ (\protect\overleftarrow{s_{1}},\protect\overleftarrow{s_{2}}), (\protect\overleftarrow{s_{1}},\protect\overrightarrow{s_{2}}), (\protect\overrightarrow{s_{1}},\protect\overleftarrow{s_{2}}), (\protect\overrightarrow{s_{1}},\protect\overrightarrow{s_{2}}) \right\}. $$

In the scaffold assembly graph, we add assembly edges encoding all realizations of semi-/un- oriented assembly points and refer to such edges as *candidate*, in contrast to *actual* assembly edges encoding oriented assembly points.

We extend the conflictedness classification to semi-oriented and unoriented assembly points as follows. An assembly point *p* is *in-conflicting* (*out-confliciting*) with an assembly point *c* if all pairs of their realizations {*p* ^′^,*c* ^′^}∈*R*(*p*)×*R*(*c*) are in-conflicting (out-confliciting). Similarly, an assembly point *p* is *in-semiconflicting* (*out-semiconfliciting*) with an assembly point *c* if some but not all pairs of their realizations are in-conflicting (out-confliciting) (Fig. 2 b, d). It is easy to see that for the case of all assembly points being oriented, this definition is consistent with the one given in the previous section.

### Merging assemblies

CAMSA can resolve conflicts in the input assemblies by merging them into a single (not self-confliciting) *merged assembly* that is most consistent with the input ones. The merged assembly is also used to determine orientation of (some) unoriented scaffolds in one input assemblies that is most confident and/or consistent with other input assemblies. In other words, the merged assembly helps to identify realizations of (some) semi-/un- oriented assembly points that are most consistent with other assemblies. Namely, for each semi-/un- oriented assembly point, the merged assembly contains either only one or none of its realizations; and in the former case, the included realization defines the most confident orientation of the corresponding unoriented scaffolds.

**Fig. 3:**
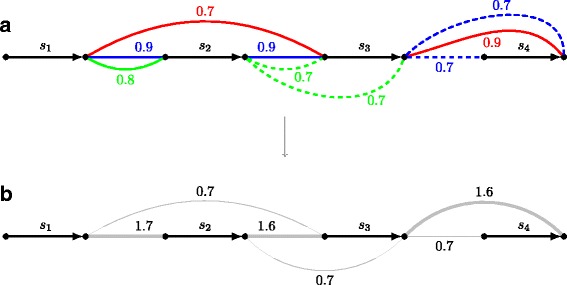
**a** Scaffold assembly graph *SAG*(*A*_1_,*A*_2_,*A*_3_), where assemblies *A*_1_, *A*_2_, and *A*_3_ are represented as red, blue, and green assembly edges, respectively, labeled with the corresponding confidence weights. **b** Merged scaffold assembly graph *MSAG*(*A*_1_,*A*_2_,*A*_3_) obtained from *SAG*(*A*_1_,*A*_2_,*A*_3_) by replacing each assembly multi-edge with an ordinary edge of combined weight. The bold assembly edges represent the merged assembly computed by CAMSA

Assembly merging performed by CAMSA is based on how often each assembly point appears in the input assemblies as well as on the (optional) confidence of each such appearance. Namely, for each assembly point *p* in an input assembly *A*, CAMSA allows to specify the *confidence weight* CW_*A*_(*p*) from the interval [ 0,1], which is then assigned to the corresponding assembly edge(s) (Fig. 3 a). The confidence weights are expected to reflect the confidence level of the assembly methods in what they report as scaffold adjacencies (e.g., heuristic methods should probably have smaller confidence as compared to more reliable wet-lab techniques). By default, all actual assembly edges have the confidence weight equal 1, and all candidate assembly edges have weight 0.75 (these default values can be overwritten by the user).

For any oriented assembly *B* (viewed as a set of oriented assembly points), we define the *consistency score* CS_*B*_(*A*) of an input assembly *A* with respect to *B* as CS_*B*_(*A*)$ = \sum _{p\in B} \overline {\textsc {cw}}_{A}(p)$, where

$$\overline{\textsc{cw}}_{A}(p) = \left\{\begin{array}{ll} 0,&\ \text{if}\ \forall\ x\in A\ :\ p\not\in R(x); \\ \textsc{cw}_{A}(x),&\ \text{if}\ \exists\ x\in A\ :\ p\in R(x). \end{array}\right. $$

We pose the *assembly merging problem* (AMP) as follows.

#### **Problem 1**

(Assembly Merging Problem, AMP) Given assemblies *A*_1_,…,*A*_*k*_ of the same set of scaffolds *S*, find an assembly *M* of *S* containing only oriented assembly points such that (i) *M* is not self-conflicting (i.e., does not contain any in-conflicting assembly points); (ii) $\sum _{i=1}^{k} \textsc {cs}_{M}(A_{i})$ is maximized; (iii) for every assembly point *p*∈*A*_1_∪⋯∪*A*_*k*_, at most one of its realizations is present in *M* (i.e., |*M*∩*R*(*p*)|≤1).

For a solution *M* to the AMP, the condition (i) implies that the assembly edges in *SAG*(*M*) form a matching. Furthermore, *M* is assumed to correspond to the genome, which may be subject to additional constraints such as having all chromosomes linear (e.g., for vertebrate genomes) or having a single chromosome (e.g., for bacterial genomes). These constraints are translated for *M* as the absence in *SAG*(*M*) of cycles formed by alternating assembly and scaffold edges (for a unichromosomal circular genome, such a cycle can be present in *SAG*(*M*) only if it includes all scaffold edges).

To address the AMP, we start with construction of the (weighted) *merged scaffold assembly graph* *MSAG*(*A*_1_,…,*A*_*k*_) from *SAG*(*A*_1_,…,*A*_*k*_) by replacing each assembly multi-edge with an ordinary assembly edge of the weight equal the total weight of the corresponding multi-edge (Fig. 3). So, *MSAG*(*A*_1_,…,*A*_*k*_) is the graph with two types of edges: unweighted directed scaffolds edges and weighted undirected assembly edges. The AMP is then can be reformulated as the following *restricted maximum matching problem* (RMMP) on the graph *G*=MSAG(*A*_1_,…,*A*_*k*_):

#### **Problem 2**

(Restricted Maximum Matching Problem, RMMP) Given a merged scaffold assembly graph *G*, find a subset *M* of assembly edges in *G* such that (i) *M* is a matching; (ii) *M* has maximum weight; (iii) there are no cycles in *SAG*(*M*).

Let *M* be a solution to the RMMP. Then the graph *SAG*(*M*) consists of scaffold edges forming a perfect matching and assembly edges from *M* forming a (possibly non-perfect) matching by the condition (i). Thus *SAG*(*M*) is formed by collection of paths and cycles, whose edges alternate between scaffold and assembly edges. Furthermore, by the condition (iii), *SAG*(*M*) consists entirely of alternating paths. A similar optimization problem, where the number of paths and the number cycles in the resulting *SAG*(*M*) are fixed, is known to be NP-complete [31], leaving a little hope for the RMMP to have a polynomial-time solution. Instead, CAMSA employs two merging heuristic solutions building upon the previously proposed algorithms [31, 32] as described below in this section.

**Greedy merging heuristics.** For a given merged scaffold assembly graph *G*, this strategy starts with the graph *H* consisting of scaffold edges from *G* and then iteratively enriches *H* with assembly edges so that no cycles are created in *H*. At any stage of this process, *H* is considered as a collection of alternating paths, some of which are merged into a longer path by adding a corresponding assembly edge. The paths to merge are selected based on the confidence weight of their linking assembly edge. The final graph *H* constructed this way defines *M* as the set of assembly edges in *H* (and so *SAG*(*M*)=*H*).

**Maximum matching heuristics.** For a given merged scaffold assembly graph *G*, this first computes the maximum weighted matching *M* ^′^ formed by assembly edges of *G*. Namely, CAMSA employs the NetworkX library [33] implementation of the blossom algorithm [34] for computing *M* ^′^.^3^ For the maximum weighted matching *M* ^′^, CAMSA looks for cycles in *SAG*(*M* ^′^) (notice that all cycles in *SAG*(*M* ^′^) are vertex-disjoint) and removes an assembly edge of the lowest confidence weight from each such cycle. These edges are also removed from *M* ^′^ to form *M* so that *SAG*(*M*) consists entirely of alternating paths.

We remark that before solving the RMMP for *G*=MSAG(*A*_1_,…,*A*_*k*_), CAMSA allows to remove assembly edges from *G* that have weight smaller than the *weight threshold* specified by the user (by default, this threshold is set to 0, i.e., no edges are removed). The removal of small-weighted assembly edges may be desirable if one wants to restrict attention only to assembly points of certain confidence level (e.g., assembly points coming either from individual highly-reliable assemblies or as a consensus from multiple assemblies). When such removal of low-confidence edges is performed, it is important to do so before (not after) solving the RMMP, since otherwise these edges may introduce a bias for an inclusion of high-confidence edges into the merged assembly *M*.

## Results

### Structure of CAMSA report

The results of comparative analysis and assembly merging performed by CAMSA are presented to the user in the form of an interactive *report*. The report is generated in a form of a JavaScript-powered HTML file, readily accessible for viewing/working in any modern Internet browser (for locally generated reports, Internet connection is not required). Many of the report sections are also available in the form of text files, making them accessible for machine processing. All tables in the report are powered by the DataTables JavaScript library [35], which provides flexible and dynamic filtering, sorting, and searching capabilities.

The first section of the CAMSA report presents aggregated characteristics of each input assemblies as compared to the others:

- the number of oriented, semi-oriented, and unoriented assembly points;
- the number of in-/out- conflicting assembly points;
- the number of in-/out- semiconflicting assembly points;
- the number of nonconflicting assembly points;
- the number of assembly points that participate in the merged assembly.

**Fig. 4:**
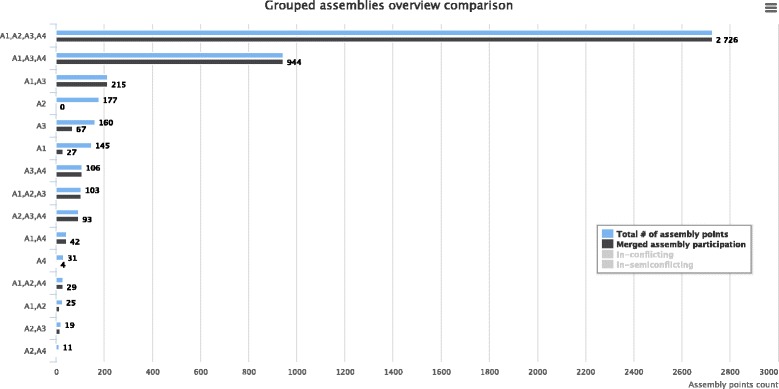
The second section of the CAMSA report for the scaffold assemblies of *H. sapiens Chr14* produced by ScaffMatch (*A*1), SGA (*A*2), SOAPdenovo2 (*A*3), and SSPACE (*A*4). For each subset of the assemblies *A*1, *A*2, *A*3, and *A*4, it gives the number of assembly points that are unique to this subset; participate in the merged assembly; are in-conflicting; and are in-semiconflicting

The second section of the CAMSA report focuses on consistency across various subsets of input assemblies. For each subset, it gives characteristics similar to the ones in the first section, but the values here are aggregated over all assemblies in the subset. The subsets are listed as a bar diagram in the descending order of the number of unique assembly points they contain (Fig. 4). Such statistics eliminates the need of running CAMSA separately on any assemblies subsets and allows the user to easily identify groups of assemblies that agree/conflict among themselves the most. We remark that each assembly point is counted only once: for the set of assemblies that contains this assembly point (but not for any of its smaller subset). Since the the number of all subsets of input assemblies can be large, CAMSA allows the user to specify the number of top subsets to be displayed.

The third section of the CAMSA report provides statistics for each assembly point within each assembly. Extensive interactive filtering allows the user to select assembly points of interest, as well as to export the filtered results, creating problem- / region- / fragment- focused analysis pipelines. We remark that statistical characteristics (e.g., whether an assembly point is in-/out- conflicting or in-/out- semiconflicting) are computed with respect to all of the assembly points in all input assemblies.

**Fig. 5:**
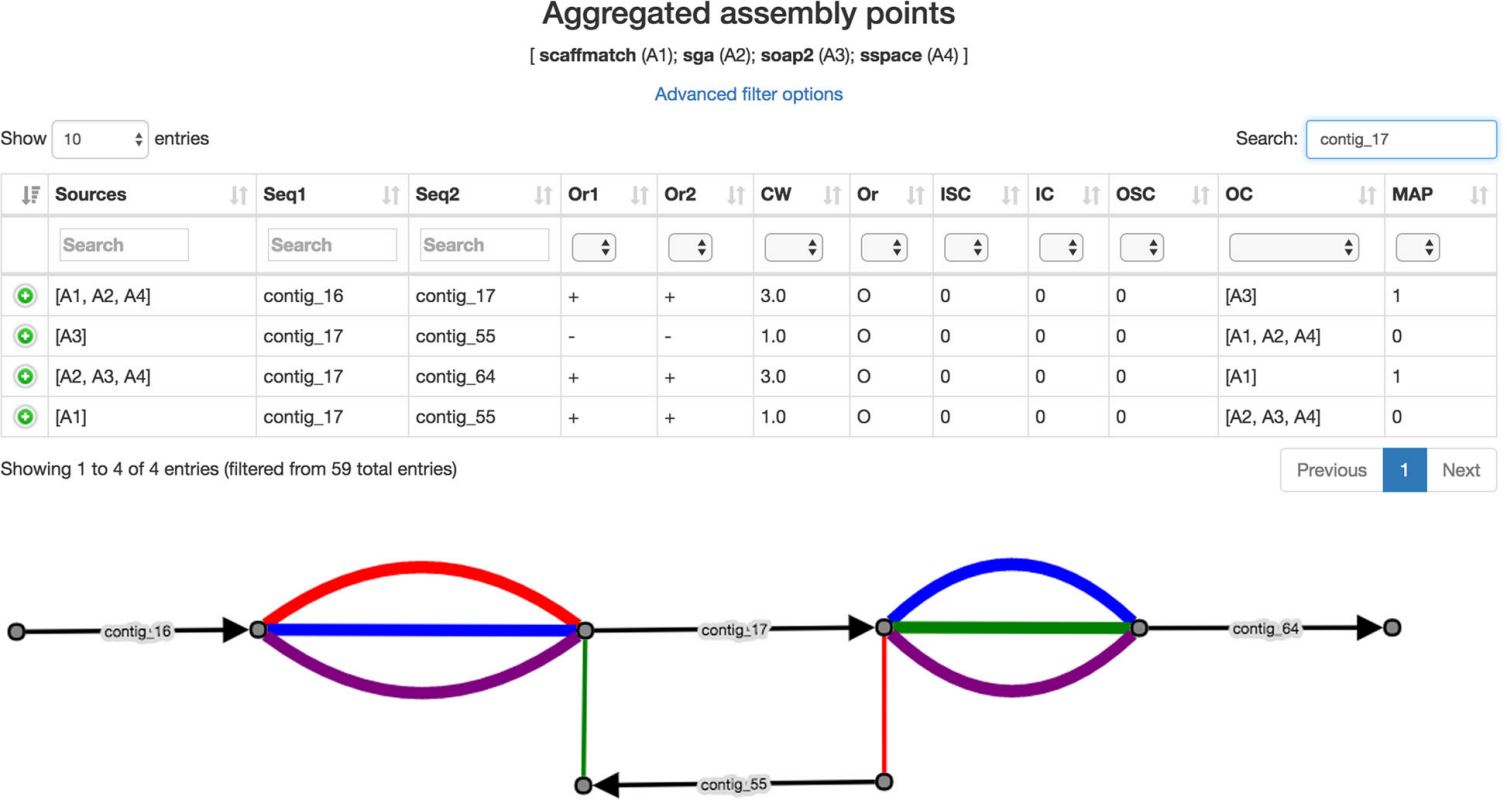
The fourth section of the CAMSA report for the scaffold assemblies of *S. aureus* produced by ScaffMatch (*A*1), SGA (*A*2), SOAPdenovo2 (*A*3), and SSPACE (*A*4). **a** Table resulting from filtration and containing only assembly points involving scaffold contig_17. **b** A subgraph of the scaffold assembly graph *SAG*(*A*1,*A*2,*A*3,*A*4) induced by the assembly points involving scaffold contig_17

The fourth section of the CAMSA report provides statistics for each assembly point aggregated over all of the input assemblies (Fig. 5). In contrast to the third section, here each assembly point is shown exactly once, and the *sources* column shows the set of assemblies where this assembly point is present (e.g., in Fig. 5 the assembly point $(\protect \overrightarrow {\texttt {contig\_16}}, \protect \overrightarrow {\texttt {contig\_17}})$ is present in *A*1, *A*2, and *A*3). Again, CAMSA provides extensive filtering to enable a focused analysis of assembly points of interest. The result of assembly points filtration can further be exported in the same format, which is utilized for CAMSA input files (i.e., list of assembly points in a tab-separated format).

Besides the text-based representation and export, the CAMSA report also provides an interactive visualization and further graphical export of assembly points in the form of the scaffold assembly graph. A vector-based interactive graph visualization is created using the Cytoscape.js library [36]. This visualization has a dynamic graph layout and supports filtration of graph components. We allow the user to choose from several Cytoscape.js graph layouts; the default layout comes from [37]. At any point the current image of the scaffold assembly graph can be exported from the report into a PNG file.

The time required for graph visualization heavily depends on the chosen layout and the underlying graph complexity. In cases when visualization inside the report takes too much time, we provide the following workarounds. The assembly points can be exported in a text file and then converted into a DOT file describing the corresponding scaffold assembly graph, whose visualization then can be constructed with GraphViz [38]. Alternatively, one can choose to export the SAG subgraph induced by the filtered assembly points into a JSON file, which can further be processed with the desktop Cytoscape software [39].

### Evaluation

While merging of multiple input scaffold assemblies is just one of the features of the CAMSA framework, it is the only one that resembles existing tools, namely those performing assembly reconciliation. We therefore feel obliged to compare its performance to such tools, even though we pose CAMSA as a meta-tool that can take as an input the results of various scaffolding methods, including assembly reconciliation tools.

We evaluated the assembly merging in CAMSA by running it on multiple scaffold assemblies of genomes of different sizes from the GAGE project [40]. While CAMSA can be used at any stage of genome scaffolding, in this evaluation we applied it to the results of initial scaffolding of contigs based on jumping libraries. We chose the following four scaffolders for performing such task: ScaffMatch [41], SOAPdenovo2 [6], SGA [42], and SSPACE [8]. The input to these scaffolders was formed by contigs and jumping libraries assembled and corrected by Allpaths-LG [43], which are provided by GAGE. The scaffold assemblies produced by these scaffolders were used as an input to CAMSA as well as to Metassembler [28] and GAM-NGS [29] assembly reconciliation tools.^4^ To demonstrate the advantages of CAMSA as a meta-tool, we also run it on the four aforementioned scaffold assemblies combined with the two reconciled assemblies produced by Metassembler and GAM-NGS, and denoted as CAMSA(+GM) in the evaluation results.

All tools were run on the same computer system with dual Intel Xeon E5-2670 2.6GHz 8-core processors and 64GB of RAM. First, we measured the running time of each tool. Then we assessed the quality of the resulting scaffold assemblies (formed by *merged scaffolds*) with the number of metrics computed by QUAST [44] with --scaffolds flag. Below we present most important metrics, while the complete QUAST reports for both input (Additional file 1: Tables S6, S7, S8) and resulting scaffold assemblies (Additional file 1: Tables S9, S10, S11) are provided in Additional file 1. Namely, we mostly consider the following QUAST metrics:

- # contigs: in our evaluation, the contigs counted by QUAST correspond the merged scaffolds; so their number measures the contiguity of the resulting scaffold assemblies.
- # misassemblies (*miss.*): number of breakpoints in the merged scaffolds, for which the left and right flanking sequences align in the reference genome to different strands / chromosomes (inversions / translocations), or on the same stand and chromosome with a gap of ≥ 1000bp between each other (relocations).
- # local misassemblies (*local miss.*): number of relocations with a gap in the range from 85bp to 1000bp.
- NA50: the maximum length *L* such that the fragments of length ≥*L* obtained from the merged scaffolds by breaking them at misassembly sites cover at least 50% of assembly.
- NA75: similar to NA50, but with 75% coverage of the assembly.

Table 1 demonstrates that CAMSA is the fastest among the tools in comparison. We separately benchmarked the data preparation and processing. We remark that depending on the format of input scaffold assemblies as well as the overall assembly pipeline, the data preparation step may be not required or take significantly different time. For CAMSA in this evaluation data preparation involves the conversion of scaffold assemblies from FASTA format into the set of assembly points,^5^ using a utility script based on NUCmer software [45] ran in parallel for each of the input assemblies. For GAM-NGS, one needs to align the jumping libraries onto the input scaffold assemblies as well as onto the intermediate reconciled assemblies (progressively generated from the input assemblies). The former alignments were treated as data preparation (since they may be readily available from the assembly pipeline), while the latter alignments are generally unavailable and thus were treated as data processing. For Metassembler, no data preparation was required since all alignments are performed internally.

**Table 1:** Running time of GAM-NGS, Metassembler, and CAMSA on scaffold assemblies produced by ScaffMatch, SOAPdenovo2, SGA, and SSPACE on three GAGE datasets

	*S. aureus*	*R. sphaeroides*	*H. sapiens Chr14*
GAM-NGS	4m25s (+2m3s)	8m47s (+4m14s)	1h29m (+43m)
Metassembler	59m16s (+0s)	1h48m53s (+0s)	8h19m10s (+0s)
CAMSA	**2s (+3s)**	**2s (+10s)**	**48s (+59m)**
CAMSA(+GM)	**2s (+3s)**	**2s (+10s)**	54s (+1h10m)

Time in parentheses is additional and corresponds to the data preparation. Best results are shown in bold

Table 2 shows the quality of the scaffold assemblies produced by different tools. In all datasets, the assembly produced by CAMSA was either the best or very close to the best in each of the metrics. We remark that in some cases CAMSA(+GM) takes advantage of the reconciled assemblies and demonstrates better results than CAMSA. In other cases, however, having the reconciled assemblies turns out to be disadvantageous due to the elevated presence of misassemblies in them. This emphasizes the fact that assembly reconciliation/merging is sensitive to the quality of input assemblies and should be interpreted with caution. The comparative report in CAMSA can greatly help in identification of conflicting assembly points (indicating potential misassemblies), enabling their targeted analysis.

**Table 2:** Quality of the reconciled/merged scaffold assemblies constructed by GAM-NGS, Metassembler, and CAMSA from the scaffold assemblies produced by ScaffMatch, SOAPdenovo2, SGA, and SSPACE on three GAGE datasets

	# contigs	# miss.	# local miss.	NA50	NA75
*S. aureus*					
GAM-NGS	**6**	**0**	6	1082860	1082860
Metassembler	**6**	**0**	3	1083010	1083010
CAMSA	**6**	**0**	3	**1083448**	**1083448**
CAMSA(+GM)	**6**	**0**	**2**	1083436	1083436
*R. sphaeroides*					
GAM-NGS	16	**6**	17	3080645	3080645
Metassembler	**9**	**6**	17	**3080845**	**3080845**
CAMSA	**9**	**6**	**9**	2965313	2965313
CAMSA(+GM)	10	**6**	**9**	2964450	2964450
*H. sapiens Chr14*					
GAM-NGS	128	**83**	543	2941846	1235019
Metassembler	**93**	94	528	2494911	1235460
CAMSA	109	91	**485**	2624904	**1235471**
CAMSA(+GM)	94	84	511	**2979834**	1235464

Best results are shown in bold

## Discussion

We remark that CAMSA expects as an input a list of assembly points, which differs from the output produced by some conventional scaffolding tools. This inspired us to develop a set of utility scripts that automate the input/output conversion process for CAMSA (e.g., from/to formats like FASTA,^6^ AGPv2, or GRIMM), and include them in the CAMSA package. We remark that our current model treats scaffolds that are present more than once in the input assembly as conflicts, thus limiting the ability to work with scaffolds coming from repetitive DNA regions. However, this issue may rarely appear for long scaffolds, and in fact we did not encounter it in our evaluations. Still, we have plans to expand the model and add support for repetitive scaffolds in future CAMSA releases.

We further plan to enrich the graph-based analysis in CAMSA with various pattern matching techniques, enabling a better classification of assembly conflicts based on their origin (e.g., conflicting scaffold orders, wrong orientation of scaffolds, or different resolution of assemblies). We also plan on adding a *reference* mode, so that classification of assembly points in the input assemblies can be done with respect to a known reference genome, rather than just with respect to each other.

## Conclusion

CAMSA addresses the current deficiency of tools for automated comparison and analysis of multiple assemblies of the same set scaffolds. Since there exist numerous methods and techniques for scaffold assembly, identifying similarities and dissimilarities across assemblies produced by different methods is beneficial both for the developers of scaffold assembly algorithms and for the researchers focused on improving draft assemblies of specific organisms.

CAMSA is currently utilized in the study of Anopheles mosquito genomes [46], where multiple research laboratories work on improving the existing assemblies for a set of mosquito species.

## Endnotes

^1^ We remark that contigs can be viewed as scaffolds with no gaps. So, under scaffolds we understand both contigs and scaffolds.

^2^ We remark that an assembly point can be in/out-conflicting with multiple different assembly points at the same time.

^3^ The blossom algorithm computes a maximal weighted matching in a graph in *O*(*V* ^3^) time, where *V* is the number of vertices.

^4^ We also considered GARM [27], but were unable to run it on any GAGE dataset, facing issues similar to those reported in [28].

^5^ We remark that conversion, for example, from NCBI AGPv2 format (rather than FASTA) would be much faster.

^6^ We support FASTA files that may or may not contain gap filling.

## Additional file

Additional file 1
CAMSA: Evaluation Details. (PDF 192 kb)
