# Supplementary material for: CAMSA: a tool for comparative analysis and merging of scaffold assemblies
Source: BMC Bioinformatics. 2017 Dec 6;18(Suppl 15):496. doi: 10.1186/s12859-017-1919-y (PMC5731503; doi:10.1186/s12859-017-1919-y)
Supplement: Additional file 1 — CAMSA: Evaluation Details. (PDF 192 kb) [file 12859_2017_1919_MOESM1_ESM.pdf]

CAMSA: Evaluation Details
(Additional file 1)

Datasets

Each dataset in the evaluation comes from the GAGE project and consists of the following files (the paths are relative to the root directory `http://gage.cbcb.umd.edu/data/<genome>/`, where `<genome>` is `Staphylococcus_aureus`, `Rhodobacter_sphaeroides`, and `Hg_chr14`, respectively):

- Reference genomic sequence file `genome.fasta` from `Data.original/` directory;
- Jumping library (corrected by Allpaths-LG) files `shortjump_{1,2}.fastq` and `longjump_{1,2}.fastq` (when available) from `Data.allpathsCor.tgz` archive;
- Contigs (assembled by Allpaths-LG) from `Assembly.tgz` archive.

Software

Data preparation, processing, and analysis in the evaluation were performed with the following software tools (particular versions are specified in parentheses):

1. Allpaths-LG (r52488) [A2] (`http://software.broadinstitute.org/allpaths-lg/blog/`)
2. QUAST (v4.1) [A3] (`http://quast.sourceforge.net/`)
3. Bowtie2 (2.2.9) [A5] (`http://bowtie-bio.sourceforge.net/bowtie2/`)
4. Picard (1.129) (`http://broadinstitute.github.io/picard`)
5. Samtools (1.2) [A6] (`http://samtools.sourceforge.net/`)
6. SGA (0.10.13) [A10] (`https://github.com/jts/sga`)
7. SOAPdenovo2 (2.04-r240) [A7] (`https://github.com/aquaskyline/SOAPdenovo2`)
8. ScaffMatch (0.9) [A8] (`http://alan.cs.gsu.edu/NGS/?q=content/scaffmatch`)
9. Abyss (1.5.2) [A11] (`https://github.com/bcgsc/abyss`)
10. SSPACE (3.0) [A1] (`http://www.baseclear.com/genomics/bioinformatics/basetools/SSPACE`)
11. Metassembler (1.5) [A13] (`https://sourceforge.net/projects/metassembler/`)
12. GAM-NGS (v1.1b) [A12] (`https://github.com/vice87/gam-ngs`)
13. GARM (0.7.5) [A9] (`http://garm-meta-assem.sourceforge.net/`)
14. CAMSA (1.0.0) (`https://cblab.org/camsa`)
15. NUCmer (3.1) [A4] (`http://mummer.sourceforge.net/`)

Experiments outline

For each GAGE dataset, the process of preparation, scaffold assembly, merging of the resulting scaffold assemblies, and their further analysis is outlined below:

1. Using QUAST, compute statistics of the Allpaths-LG contigs (Supplementary Tables S3, S4, S5).
2. Using Bowtie2, align the Allpaths-LG corrected shortjump (and longjump, when available) jumping libraries to the reference genome sequence.
3. Using Samtools and Picard tools, from the obtained reads-to-reference alignment determine jumping library orientation, the median insert size and its standard deviation (Supplementary Table S1).
4. (when required) Using Bowtie2, align the same corrected jumping libraries to the Allpaths-LG contigs (Supplementary Table S2 describes the alignment parameters).
5. Scaffold the Allpaths-LG contigs with different scaffolders, using shortjump (and longjump, when available) jumping libraries.
6. Using QUAST, compute statistics of the obtained scaffold assemblies (Supplementary Tables S6, S7, S8).
7. Merge the obtained scaffold assemblies, using CAMSA, Metassembler, and GAM-NGS.
8. Using QUAST, compute statistics of the obtained merged scaffold assemblies (Supplementary Tables S9, S10, S11).

Table S1: Metrics computed with Picard's `CollectInsertSizeMetrics` tool for jumping libraries in the three observed GAGE datasets.

Dataset	Library	Orientation	Median insert size	Standard deviation	Size
S. aureus	shortjump	RF	3609	265	475408
R. sphaeroides	shortjump	RF	3761	716	516804
		FR	344	97	34296
H. sapiens Chr14	shortjump	RF	2702	256	2095666
		FR	242	90	706331
	longjump	FR	34755	7892	80331

Table S2: Bowtie2 parameters used for alignment of jumping libraries onto Allpaths-LG assembled contigs in the three observed GAGE dataset.

Dataset	Library	Bowtie2 parameters
S. aureus	shortjump	`--minins 3300 --maxins 3900 --rf`
R. sphaeroides	shortjump	`--minins 3000 --maxins 4450 --rf`
H. sapience Chr14	shortjump	`--minins 2400 --maxins 3000 --rf`
	longjump	`--minins 27000 --maxins 42500`

Table S3: QUAST report for the contigs of *S. aureus* assembled by Allpaths-LG.

Assembly	*S. aureus*
# contigs ($\geq$ 1000 bp)	58
# contigs ($\geq$ 5000 bp)	45
# contigs ($\geq$ 10000 bp)	40
# contigs ($\geq$ 25000 bp)	35
# contigs ($\geq$ 50000 bp)	19
Total length ($\geq$ 0 bp)	2870776
Total length ($\geq$ 1000 bp)	2868733
Total length ($\geq$ 5000 bp)	2840918
Total length ($\geq$ 10000 bp)	2800923
Total length ($\geq$ 25000 bp)	2715192
Total length ($\geq$ 50000 bp)	2129621
# contigs	59
Largest contig	234488
Total length	2869581
Reference length	2903081
GC (%)	32.65
Reference GC (%)	32.73
N50	96740
NG50	96740
N75	48304
NG75	48304
L50	10
LG50	10
L75	20
LG75	20
# misassemblies	0
# misassembled contigs	0
Misassembled contigs length	0
# local misassemblies	0
# unaligned contigs	0 + 0 part
Unaligned length	0
Genome fraction (%)	98.818
Duplication ratio	1.000
# N's per 100 kbp	1.50
# mismatches per 100 kbp	1.92
# indels per 100 kbp	1.01
Largest alignment	234488
NA50	96740
NGA50	96740
NA75	45922
NGA75	45922
LA50	10
LGA50	10
LA75	21
LGA75	21

Table S4: QUAST report for the contigs of *R. sphaeroides* assembled by Allpaths-LG.

Assembly	*R. sphaeroides*
# contigs (≥ 1000 bp)	202
# contigs (≥ 5000 bp)	151
# contigs (≥ 10000 bp)	123
# contigs (≥ 25000 bp)	67
# contigs (≥ 50000 bp)	26
Total length (≥ 0 bp)	4588376
Total length (≥ 1000 bp)	4586421
Total length (≥ 5000 bp)	4448086
Total length (≥ 10000 bp)	4241155
Total length (≥ 25000 bp)	3290635
Total length (≥ 50000 bp)	1794597
# contigs	203
Largest contig	106467
Total length	4587354
Reference length	4603060
GC (%)	68.73
Reference GC (%)	68.79
N50	42455
NG50	42455
N75	23323
NG75	23323
L50	37
LG50	37
L75	74
LG75	74
# misassemblies	5
# misassembled contigs	3
Misassembled contigs length	183375
# local misassemblies	2
# unaligned contigs	0 + 1 part
Unaligned length	92
Genome fraction (%)	99.292
Duplication ratio	1.004
# N's per 100 kbp	2.79
# mismatches per 100 kbp	6.15
# indels per 100 kbp	4.68
Largest alignment	105281
NA50	41334
NGA50	41334
NA75	20202
NGA75	20202
LA50	39
LGA50	39
LA75	80
LGA75	80

Table S5: QUAST report for the contigs of *H. sapiens Chr14* assembled by Allpaths-LG.

Assembly	*H. Sapiens Chr14*
# contigs (≥ 1000 bp)	4383
# contigs (≥ 5000 bp)	2965
# contigs (≥ 10000 bp)	2256
# contigs (≥ 25000 bp)	1152
# contigs (≥ 50000 bp)	407
Total length (≥ 0 bp)	84461065
Total length (≥ 1000 bp)	84346908
Total length (≥ 5000 bp)	80952335
Total length (≥ 10000 bp)	75700427
Total length (≥ 25000 bp)	57826694
Total length (≥ 50000 bp)	31727320
# contigs	4469
Largest contig	240773
Total length	84416102
Reference length	107349540
GC (%)	40.77
Reference GC (%)	40.89
N50	38359
NG50	27960
N75	20286
NG75	5549
L50	646
LG50	995
L75	1396
LG75	2882
# misassemblies	51
# misassembled contigs	51
Misassembled contigs length	594366
# local misassemblies	138
# unaligned contigs	0 + 78 part
Unaligned length	10377
Genome fraction (%)	78.466
Duplication ratio	1.002
# N's per 100 kbp	54.60
# mismatches per 100 kbp	67.25
# indels per 100 kbp	21.79
Largest alignment	240773
NA50	38186
NGA50	27586
NA75	20019
NGA75	5273
LA50	647
LGA50	999
LA75	1404
LGA75	2917

Table S6: QUAST report for 4 obtained scaffold assemblies on the *S. aureus* dataset.

Assembly	SGA	SSPACE	SOAPdenovo2	ScaffMatch
# contigs ($\geq$ 1000 bp)	16	8	**6**	7
# contigs ($\geq$ 5000 bp)	10	**6**	**6**	**6**
# contigs ($\geq$ 10000 bp)	7	**6**	**6**	**6**
# contigs ($\geq$ 25000 bp)	7	6	**5**	**5**
# contigs ($\geq$ 50000 bp)	6	**4**	**4**	**4**
Total length ($\geq$ 0 bp)	2879803	**2888431**	2885823	2885463
Total length ($\geq$ 1000 bp)	2879515	**2887236**	2884916	2884556
Total length ($\geq$ 5000 bp)	2867655	2884858	**2884916**	2883180
Total length ($\geq$ 10000 bp)	2847154	2884858	**2884916**	2883180
Total length ($\geq$ 25000 bp)	2847154	**2884858**	2860116	2859903
Total length ($\geq$ 50000 bp)	2808847	2820425	**2821809**	2821596
# contigs	16	8	**6**	7
Largest contig	1436473	**1437245**	1436067	1436757
Total length	2879515	**2887236**	2884916	2884556
Reference length	2903081	2903081	2903081	2903081
GC (%)	32.65	32.65	32.65	32.65
Reference GC (%)	32.73	32.73	32.73	32.73
N50	690085	1093113	**1096127**	1094991
NG50	690085	1093113	**1096127**	1094991
N75	231306	1093113	**1096127**	1094991
NG75	231306	1093113	**1096127**	1094991
L50	**2**	**2**	**2**	**2**
LG50	**2**	**2**	**2**	**2**
L75	3	**2**	**2**	**2**
LG75	3	**2**	**2**	**2**
# misassemblies	**0**	**0**	**0**	**0**
# misassembled contigs	**0**	**0**	**0**	**0**
Misassembled contigs length	**0**	**0**	**0**	**0**
# local misassemblies	**1**	2	6	3
# scaffold gap size misassemblies	**23**	41	28	31
# unaligned contigs	**0 + 0 part**	**0 + 0 part**	**0 + 0 part**	**0 + 0 part**
Unaligned length	**0**	**0**	**0**	**0**
Genome fraction (%)	98.832	98.824	98.817	**98.847**
Duplication ratio	**1.004**	1.006	1.006	1.005
# N's per 100 kbp	**368.29**	616.26	568.37	510.75
# mismatches per 100 kbp	3.83	3.97	**3.17**	4.46
# indels per 100 kbp	1.92	**1.85**	2.41	2.93
Largest alignment	**1394578**	1394477	1373048	1393974
NA50	685027	1080969	1082860	**1083369**
NGA50	685027	1080969	1082860	**1083369**
NA75	231127	1080969	1082860	**1083369**
NGA75	231127	1080969	1082860	**1083369**
LA50	**2**	**2**	**2**	**2**
LGA50	**2**	**2**	**2**	**2**
LA75	3	**2**	**2**	**2**
LGA75	3	**2**	**2**	**2**

Table S7: QUAST report for 4 obtained scaffold assemblies on the *R. sphaeroides* dataset.

Assembly	SGA	SSPACE	SOAPdenovo2	ScaffMatch
# contigs ($\geq$ 1000 bp)	79	**15**	16	16
# contigs ($\geq$ 5000 bp)	55	9	**8**	9
# contigs ($\geq$ 10000 bp)	50	9	**8**	**8**
# contigs ($\geq$ 25000 bp)	39	8	**7**	**7**
# contigs ($\geq$ 50000 bp)	23	7	**6**	**6**
Total length ($\geq$ 0 bp)	4600980	4609201	4599261	**4619707**
Total length ($\geq$ 1000 bp)	4600506	4607246	4598239	**4619159**
Total length ($\geq$ 5000 bp)	4551065	4598883	4586030	**4607565**
Total length ($\geq$ 10000 bp)	4515666	**4598883**	4586030	4598709
Total length ($\geq$ 25000 bp)	4350981	4584344	4571440	**4586110**
Total length ($\geq$ 50000 bp)	3784532	4550576	4537672	**4552342**
# contigs	79	**16**	**16**	**16**
Largest contig	471741	1605102	**3186031**	2544306
Total length	4600506	4608179	4598239	**4619159**
Reference length	4603060	4603060	4603060	4603060
GC (%)	68.72	68.73	68.72	68.73
Reference GC (%)	68.79	68.79	68.79	68.79
N50	211952	1581585	**3186031**	2544306
NG50	211952	1581585	**3186031**	2544306
N75	73878	**912982**	911802	654158
NG75	73878	912982	911802	**914068**
L50	8	2	**1**	**1**
LG50	8	2	**1**	**1**
L75	18	3	**2**	3
LG75	18	3	**2**	**2**
# misassemblies	**5**	6	6	6
# misassembled contigs	3	3	3	**2**
Misassembled contigs length	**183375**	335048	324030	323930
# local misassemblies	**3**	11	17	8
# scaffold gap size misassemblies	**36**	45	44	56
# unaligned contigs	**0 + 0 part**	**0 + 0 part**	**0 + 0 part**	0 + 1 part
Unaligned length	**0**	**0**	**0**	4634
Genome fraction (%)	99.318	99.338	99.305	**99.342**
Duplication ratio	**1.006**	1.008	**1.006**	1.009
# N's per 100 kbp	**336.16**	470.88	338.39	681.05
# mismatches per 100 kbp	6.41	5.88	7.79	**4.90**
# indels per 100 kbp	**6.67**	7.35	8.16	7.22
Largest alignment	450352	1522122	**3080645**	2356266
NA50	186972	1477526	**3080645**	2356266
NGA50	186972	1477526	**3080645**	2356266
NA75	58483	**907420**	886493	609106
NGA75	58483	**907420**	886493	609106
LA50	9	2	**1**	**1**
LGA50	9	2	**1**	**1**
LA75	21	3	**2**	3
LGA75	21	3	**2**	3

Table S8: QUAST report for 4 obtained scaffold assemblies on the *H. sapiens Chr14* dataset.

Assembly	SSPACE	SOAPdenovo2	ScaffMatch	SGA
# contigs ($\geq$ 1000 bp)	469	**116**	231	1238
# contigs ($\geq$ 5000 bp)	358	**50**	80	759
# contigs ($\geq$ 10000 bp)	331	**33**	51	558
# contigs ($\geq$ 25000 bp)	288	**16**	36	339
# contigs ($\geq$ 50000 bp)	246	**14**	33	283
Total length ($\geq$ 0 bp)	86098535	87702314	87845451	**88055393**
Total length ($\geq$ 1000 bp)	86032471	87659994	87786387	**87998362**
Total length ($\geq$ 5000 bp)	85823605	**87532714**	87507509	86938901
Total length ($\geq$ 10000 bp)	85623892	**87413294**	87301150	85463731
Total length ($\geq$ 25000 bp)	84949206	**87150404**	87074047	82203105
Total length ($\geq$ 50000 bp)	83421289	**87082426**	86957082	80276434
# contigs	505	**131**	257	1288
Largest contig	3247728	**27431299**	11818947	1527937
Total length	86060510	87671707	87807154	**88037622**
Reference length	107349540	107349540	107349540	107349540
GC (%)	40.77	40.78	40.77	40.77
Reference GC (%)	40.89	40.89	40.89	40.89
N50	479683	**16717346**	6850065	388109
NG50	383690	**12499179**	4587587	296840
N75	267450	**9680384**	2625940	178997
NG75	92980	**2923951**	1342148	48481
L50	50	**2**	5	67
LG50	75	**3**	7	95
L75	108	**4**	10	148
LG75	204	**7**	17	288
# misassemblies	**63**	82	135	73
# misassembled contigs	42	**18**	26	60
Misassembled contigs length	20029844	83944488	80703080	**15643725**
# local misassemblies	**460**	540	543	292
# scaffold gap size misassemblies	2647	3649	2680	**2200**
# unaligned contigs	0 + 4 part	**0 + 3 part**	0 + 4 part	0 + 24 part
Unaligned length	**2008**	3964	11617	5680
Genome fraction (%)	78.492	78.387	**78.496**	78.459
Duplication ratio	**1.021**	1.042	1.042	1.045
# N's per 100 kbp	**1960.83**	3823.84	3906.84	4188.37
# mismatches per 100 kbp	68.21	119.21	**67.34**	68.82
# indels per 100 kbp	23.21	**22.00**	23.25	22.94
Largest alignment	1734949	**6288691**	3518880	1493355
NA50	414636	**2941846**	1399071	326554
NGA50	333185	**1965866**	1001085	227608
NA75	215879	**1418204**	554190	126637
NGA75	59495	**346413**	155068	13585
LA50	58	**11**	22	76
LGA50	87	**16**	31	111
LA75	127	**23**	48	184
LGA75	251	**46**	96	497

Table S9: QUAST report for 4 merged scaffold assemblies on the *S. aureus* dataset.

Assembly	Metassembler	GAM-NGS	CAMSA	CAMSA (+GM)
# contigs (≥ 1000 bp)	**6**	**6**	**6**	**6**
# contigs (≥ 5000 bp)	**6**	**6**	**6**	**6**
# contigs (≥ 10000 bp)	**6**	**6**	**6**	**6**
# contigs (≥ 25000 bp)	**5**	**5**	6	6
# contigs (≥ 50000 bp)	**4**	**4**	**4**	**4**
Total length (≥ 0 bp)	2886309	2885823	2887925	**2889036**
Total length (≥ 1000 bp)	2886309	2884916	2886730	**2888129**
Total length (≥ 5000 bp)	2886309	2884916	2886730	**2888129**
Total length (≥ 10000 bp)	2886309	2884916	2886730	**2888129**
Total length (≥ 25000 bp)	2861509	2860116	2886730	**2888129**
Total length (≥ 50000 bp)	2823202	2821809	2822351	**2823801**
# contigs	**6**	**6**	**6**	**6**
Largest contig	1436492	1436067	1436593	**1437308**
Total length	2886309	2884916	2886730	**2888129**
Reference length	2903081	2903081	2903081	2903081
GC (%)	32.65	32.65	32.65	32.65
Reference GC (%)	32.73	32.73	32.73	32.73
N50	**1096942**	1096127	1095842	1096621
NG50	**1096942**	1096127	1095842	1096621
N75	**1096942**	1096127	1095842	1096621
NG75	**1096942**	1096127	1095842	1096621
L50	**2**	**2**	**2**	**2**
LG50	**2**	**2**	**2**	**2**
L75	**2**	**2**	**2**	**2**
LG75	**2**	**2**	**2**	**2**
# misassemblies	**0**	**0**	**0**	**0**
# misassembled contigs	**0**	**0**	**0**	**0**
Misassembled contigs length	**0**	**0**	**0**	**0**
# local misassemblies	3	6	3	**2**
# scaffold gap size misassemblies	31	**28**	35	36
# unaligned contigs	**0 + 0 part**	**0 + 0 part**	**0 + 0 part**	**0 + 0 part**
Unaligned length	**0**	**0**	**0**	**0**
Genome fraction (%)	98.822	98.817	98.833	**98.847**
Duplication ratio	**1.006**	**1.006**	**1.006**	1.007
# N's per 100 kbp	607.70	**568.37**	595.55	633.84
# mismatches per 100 kbp	4.15	**3.17**	3.66	4.53
# indels per 100 kbp	2.37	2.41	**2.27**	2.61
Largest alignment	1374235	1373048	1373608	**1394118**
NA50	1083010	1082860	**1083448**	1083436
NGA50	1083010	1082860	**1083448**	1083436
NA75	1083010	1082860	**1083448**	1083436
NGA75	1083010	1082860	**1083448**	1083436
LA50	**2**	**2**	**2**	**2**
LGA50	**2**	**2**	**2**	**2**
LA75	**2**	**2**	**2**	**2**
LGA75	**2**	**2**	**2**	**2**

Table S10: QUAST report for 4 merged scaffold assemblies on the *R. sphaeroides* dataset.

Assembly	Metassembler	GAM-NGS	CAMSA	CAMSA (+GM)
# contigs (≥ 1000 bp)	**9**	16	**9**	10
# contigs (≥ 5000 bp)	**8**	**8**	**8**	**8**
# contigs (≥ 10000 bp)	**7**	8	**7**	**7**
# contigs (≥ 25000 bp)	**6**	7	**6**	**6**
# contigs (≥ 50000 bp)	**5**	6	**5**	**5**
Total length (≥ 0 bp)	4611278	4599261	**4619613**	4618974
Total length (≥ 1000 bp)	4610804	4598239	**4618591**	4617952
Total length (≥ 5000 bp)	4609674	4586030	**4617443**	4615674
Total length (≥ 10000 bp)	4600818	4586030	**4608585**	4606816
Total length (≥ 25000 bp)	4586228	4571440	**4593951**	4592182
Total length (≥ 50000 bp)	4552460	4537672	**4560183**	4558414
# contigs	**9**	16	**9**	10
Largest contig	3186910	3186031	**3193211**	3191922
Total length	4610804	4598239	**4618591**	4617952
Reference length	4603060	4603060	4603060	4603060
GC (%)	68.72	68.72	68.73	68.73
Reference GC (%)	68.79	68.79	68.79	68.79
N50	3186910	3186031	**3193211**	3191922
NG50	3186910	3186031	**3193211**	3191922
N75	912794	911802	**913444**	913317
NG75	912794	911802	**913444**	913317
L50	**1**	**1**	**1**	**1**
LG50	**1**	**1**	**1**	**1**
L75	**2**	**2**	**2**	**2**
LG75	**2**	**2**	**2**	**2**
# misassemblies	**6**	**6**	**6**	**6**
# misassembled contigs	**2**	3	**2**	**2**
Misassembled contigs length	336947	**324030**	337651	337303
# local misassemblies	17	17	**9**	**9**
# scaffold gap size misassemblies	47	**44**	58	58
# unaligned contigs	0 + 1 part	**0 + 0 part**	0 + 1 part	0 + 1 part
Unaligned length	4634	**0**	4636	4636
Genome fraction (%)	99.317	99.305	99.335	**99.337**
Duplication ratio	1.008	**1.006**	1.009	1.009
# N's per 100 kbp	621.87	**338.39**	679.10	665.36
# mismatches per 100 kbp	7.77	7.77	**4.42**	**4.42**
# indels per 100 kbp	8.16	8.16	**7.28**	7.33
Largest alignment	**3080845**	3080645	2964686	2964450
NA50	**3080845**	3080645	2964686	2964450
NGA50	**3080845**	3080645	2964686	2964450
NA75	886881	886493	**907644**	907562
NGA75	886881	886493	**907644**	907562
LA50	**1**	**1**	**1**	**1**
LGA50	**1**	**1**	**1**	**1**
LA75	**2**	**2**	**2**	**2**
LGA75	**2**	**2**	**2**	**2**

Table S11: QUAST report for 4 merged scaffold assemblies on the *H. sapience Chr14* dataset.

Assembly	Metassembler	GAM-NGS	CAMSA	CAMSA (+GM)
# contigs (≥ 1000 bp)	86	113	94	**82**
# contigs (≥ 5000 bp)	39	48	37	**36**
# contigs (≥ 10000 bp)	25	30	26	**24**
# contigs (≥ 25000 bp)	13	16	17	**12**
# contigs (≥ 50000 bp)	12	14	16	**11**
Total length (≥ 0 bp)	87389910	87446601	87600310	**87616405**
Total length (≥ 1000 bp)	87380181	87404281	87552837	**87571603**
Total length (≥ 5000 bp)	87288768	87279334	87445013	**87477118**
Total length (≥ 10000 bp)	87192623	87149702	87370005	**87392787**
Total length (≥ 25000 bp)	87012955	86929915	**87228898**	87210713
Total length (≥ 50000 bp)	86983336	86861937	**87199315**	87181114
# contigs	**93**	128	109	94
Largest contig	27384426	**27431299**	22482944	27424679
Total length	87385570	87415994	87565052	**87581387**
Reference length	107349540	107349540	107349540	107349540
GC (%)	40.78	40.79	40.77	40.77
Reference GC (%)	40.89	40.89	40.89	40.89
N50	22403791	16717346	13553269	**22473965**
NG50	12475515	12499179	10575908	**15417218**
N75	9667993	9680384	9654501	**9681009**
NG75	3752984	2142674	3315947	**5601426**
L50	**2**	**2**	**2**	**2**
LG50	**3**	**3**	**3**	**3**
L75	**4**	**4**	**4**	**4**
LG75	6	8	7	**5**
# misassemblies	94	**83**	91	84
# misassembled contigs	**12**	18	18	13
Misassembled contigs length	85384146	83686313	**78882289**	85586152
# local misassemblies	528	543	**485**	511
# scaffold gap size misassemblies	**2964**	3639	3225	3303
# unaligned contigs	**0 + 2 part**	0 + 3 part	0 + 3 part	**0 + 2 part**
Unaligned length	**3716**	3964	8500	5087
Genome fraction (%)	78.449	78.161	**78.486**	78.485
Duplication ratio	**1.038**	1.042	1.039	1.040
# N's per 100 kbp	**3470.36**	3820.26	3629.33	3655.38
# mismatches per 100 kbp	119.28	119.05	67.82	**67.71**
# indels per 100 kbp	23.19	**22.01**	22.54	22.39
Largest alignment	6291358	6288691	5567517	**6299514**
NA50	2494911	2941846	2624904	**2979834**
NGA50	1836234	1836096	2164762	**2182939**
NA75	1235460	1235019	**1235471**	1235464
NGA75	272697	**337612**	272875	327733
LA50	12	**11**	13	**11**
LGA50	17	16	17	**14**
LA75	25	24	24	**21**
LGA75	51	48	50	**46**

Additional References

[A1] M. Boetzer, C. V. Henkel, H. J. Jansen, D. Butler, and W. Pirovano. "Scaffolding pre-assembled contigs using SSPACE". *Bioinformatics* 27:4 (2011), pp. 578–579.

[A2] S. Gnerre, I. MacCallum, D. Przybylski, F. J. Ribeiro, J. N. Burton, et al. "High-quality draft assemblies of mammalian genomes from massively parallel sequence data". *Proceedings of the National Academy of Sciences* 108:4 (2011), pp. 1513–1518.

[A3] A. Gurevich, V. Saveliev, N. Vyahhi, and G. Tesler. "QUAST: quality assessment tool for genome assemblies". *Bioinformatics* 29:8 (2013), pp. 1072–1075.

[A4] S. Kurtz, A. Phillippy, A. L. Delcher, M. Smoot, M. Shumway, et al. "Versatile and open software for comparing large genomes". *Genome Biology* 5:2 (2004), R12.

[A5] B. Langmead and S. L. Salzberg. "Fast gapped-read alignment with Bowtie 2". *Nature Methods* 9:4 (2012), pp. 357–359.

[A6] H. Li, B. Handsaker, A. Wysoker, T. Fennell, J. Ruan, et al. "The sequence alignment/map format and SAMtools". *Bioinformatics* 25:16 (2009), pp. 2078–2079.

[A7] R. Luo, B. Liu, Y. Xie, Z. Li, W. Huang, et al. "SOAPdenovo2: an empirically improved memory-efficient short-read de novo assembler". *GigaScience* 1 (2012), p. 18.

[A8] I. Mandric and A. Zelikovsky. "ScaffMatch: scaffolding algorithm based on maximum weight matching". *Bioinformatics* 31:16 (2015), pp. 2632–2638.

[A9] L. Mayela Soto-Jimenez, K. Estrada, and A. Sanchez-Flores. "GARM: genome assembly, reconciliation and merging pipeline". *Current topics in medicinal chemistry* 14:3 (2014), pp. 418–424.

[A10] J. T. Simpson and R. Durbin. "Efficient de novo assembly of large genomes using compressed data structures". *Genome Research* 22:3 (2012), pp. 549–556.

[A11] J. T. Simpson, K. Wong, S. D. Jackman, J. E. Schein, S. J. Jones, et al. "ABySS: a parallel assembler for short read sequence data". *Genome Research* 19:6 (2009), pp. 1117–1123.

[A12] R. Vicedomini, F. Vezzi, S. Scalabrin, L. Arvestad, and A. Policriti. "GAM-NGS: genomic assemblies merger for next generation sequencing". *BMC Bioinformatics* 14:Suppl 7 (2013), S6.

[A13] A. H. Wences and M. C. Schatz. "Metassembler: merging and optimizing de novo genome assemblies". *Genome Biology* 16 (2015), p. 207.
